# Psychosocial Perspectives and the Issue of Prevention in Childhood Obesity

**DOI:** 10.3389/fpubh.2014.00104

**Published:** 2014-07-31

**Authors:** Daniel Stein, Sarah L. Weinberger-Litman, Yael Latzer

**Affiliations:** ^1^Pediatric Psychosomatic Department, Edmond and Lily Safra Children’s Hospital, Chaim Sheba Medical Center, Tel Hashomer, Israel; ^2^Sackler Faculty of Medicine, Tel Aviv University, Tel Aviv, Israel; ^3^Department of Psychology, Marymount Manhattan College, New York, NY, USA; ^4^Faculty of Social Welfare and Health Sciences, Haifa University, Haifa, Israel; ^5^Eating Disorders Clinic, Psychiatric Division, Rambam Medical Center, Haifa, Israel

**Keywords:** adolescence, childhood, family, media, obesity, overweight prevention, psychosocial

## Abstract

A dramatic increase in childhood overweight/obesity has been recognized globally over the past 50 years. This observed increase may reflect genetic, as well as psychological, environmental, and socio-cultural influences. In the first part of this review, we present an updated summary of the psychosocial factors associated with this change and discuss possible ways in which they operate. Among these factors, lower socio economic status (in both industrialized and non-industrialized countries), being female, belonging to a minority group, and being exposed to adverse life events may all be associated with a greater risk of childhood overweight/obesity. These influences may be mediated via a variety of mechanisms, in particular above-average food intake of low nutritional quality and reduction in physical activity. Other important psychosocial mediators include the influence of the family and peer environment, and exposure to the media. In the second part of the review, we discuss the potential of psychosocial prevention programs to intervene in the processes involved in the rise of childhood overweight/obesity. Two points are emphasized. First, prevention programs should be multidisciplinary, combining the knowledge of experts from different professions, and taking into consideration the important role of the family environment and relevant influential social organizations, particularly school. Second, effective change is unlikely to occur without large-scale programs carried out on a public policy level.

## Introduction

There has been a dramatic global increase in childhood obesity in the past 50 years. Combating childhood overweight/obesity is one of the major public health issues facing industrialized and developing nations (1). Extensive research and large-scale campaigns designed to reduce overweight/obesity in children and adolescents have yielded little success (2). Therefore, considerable effort has been placed on identifying risk factors in order to target appropriate interventions. The etiology of obesity is multi-faceted and complex, involving both psychosocial (3) and genetic (4) factors. Although the rapid increase in global obesity is largely influenced by psychosocial factors (3), genetic factors and gene-by-environment interaction are also influential (4). In his excellent recent review, Speakman (4) argues that there is currently no consensus as to which genetic model best explains the evolutionary context of the obesity epidemic. Still he suggests that modern genetic techniques may allow a direct estimate of whether particular genes enabling efficient storage of energy as fat, likely increasing the risk for obesity, are under recent strong selection. Alternatively, obesity might have increased in recent decades as a maladaptive by-product of positive selection on some other trait (4).

The investigation of the psychosocial aspects of childhood overweight/obesity has been the focus of long-standing theoretical and empirical endeavors (5, 6). Thus, the aim of this paper is to present the main psychosocial factors associated with increased risk for overweight and/or obesity in children and adolescents and to summarize the psychosocial-related strategies for prevention. The review is based on a comprehensive literature search of the MEDLINE, PUBMED, PSYCINFO, ERIC, EMBASE, and Cochrane databases carried out between 1991 and 2013 using the following terms: obesity, overweight, risk for overweight, childhood, pediatric, adolescence, youngster, age, gender, psychosocial, socio-economic status (SES), feeding, eating, diet, physical activity, adverse life events, abuse, family, peer, kindergarten, school, prevention, primary, secondary, advertisement, media, marketing, psychoeducation, and public policy.

Obesity is a well-defined term in adults but in children and adolescents its definition is less consistent (7). The most commonly accepted definition of obesity, among individuals 2–19 years old, is that of the Center for Disease Control and Prevention, and is defined as the 95th percentile or greater of body mass index (BMI) – for age and gender. Overweight is defined as the 85th percentile or greater, but less than the 95th percentile of BMI – for age and gender. Because of the likelihood of stigmatization associated with the term “obesity,” several leading authorities have suggested replacing the term obesity with overweight, defined as BMI above the 95th percentile, and “risk for overweight” as BMI between the 85th and 95th percentile for age and gender [see Ref. (8, 9)]. Finally, an international forum of consensus development, the International Obesity Task Force (IOTF) (10), has defined childhood overweight as a BMI of approximately the 91st percentile or above and obesity as a BMI of the 99th percentile or greater for age and gender (11, 12).

## Psychosocial Risk Factors

### Socio-economic status

Research has consistently indicated an inverse relationship between SES and obesity among adults in industrialized and non-industrialized nations (3, 13, 14). Similarly, lower levels of education are associated with higher rates of obesity.

The findings are less clear in younger ages. Most studies in industrialized countries have indicated a similar relationship between low SES and higher levels of overweight/obesity (15–18), particularly among children of minority groups (19–23). This trend is also the case in most (17, 24, 25) although not all (26) studies assessing other less affluent populations.

Still, other studies suggest that among children of higher SES, overweight and obesity are directly related to the number of hours that mothers spent working outside the home (23, 27), likely interfering with their children’s access to healthy foods and physical activity. Finally, other studies have found no association between childhood overweight/obesity and SES (13).

In most studies among developing nations (23, 28, 29), the elevated risk of overweight/obesity in children of lower SES status mirrors adult findings. Several factors have been suggested to account for this finding. First and foremost, the rise in the rate of childhood overweight/obesity is most prevalent in cultures considered to be in transition (14), i.e., experiencing considerable changes within a relatively brief period of time, as is the case with many other physical and emotional disorders (30). Elevated rates of childhood overweight/obesity in developing countries and less affluent populations in industrialized countries have been associated with an inclination toward overfeeding of children or limitation of feeding (21, 31–33), above-average levels of dietary intake of low nutritional quality (31, 32, 34) and high-fat high-calorie foods and beverages (21, 32, 34), inadequate physical activity, and a greater amount of time spent in sedentary behaviors (25, 34), as well as with decreased consumption of vegetables (21, 25, 31–36). Impoverished environments with limited availability of healthful foods, higher than average availability of fast-food restaurants, and exposure to ethnically targeted food marketing (35) may all contribute to higher overweight/obesity among children (24, 37).

From a different perspective, low-income mothers of overweight pre-school children may use food to cope with the stresses of their economic situation, have difficulty setting their children limits regarding food, and are less committed to sustained behavioral change than high-income mothers (38). Moreover, food may be used in impoverished surroundings to meet emotional needs in childhood, directing overeating to become a generalized response to negative emotional states in later years (39). As a result, experiences of poverty-associated food deprivation during childhood may motivate some women to actively avoid food insecurity in adulthood, both with respect to themselves and their children (39). This association between childhood food insecurity and increased risk for overweight remains significant after controlling for ethnicity, gender, age, and family poverty level (24).

According to a 2006 review (40), at least 30% of the calories in the average diet of an American child derive from sweets, soft drinks, salty snacks, and fast food. Soft drinks account for more than 10% of the caloric intake, representing a doubling since 1980. One large-scale study in Canada found that children report preference for portions of French fries, meat, and potato chips that are larger, and for vegetable portions that are smaller, than what is recommended. Children from socio-economically disadvantaged families, or those who frequently eat while watching television and in fast-food restaurants, prefer larger portions of French fries and potato chips. Consequences of consuming large portions of these foods have been found to include poor diet quality and increased energy intake, whereas the opposite findings are shown when consuming large portions of vegetables (32). Although most of these findings have been documented in industrialized nations, similar trends have been observed also in less industrialized countries (20, 21).

In addition to SES, age, gender, and cultural background are considered as particularly influential in the recent dramatic rise in childhood overweight/obesity. These factors have been shown to be particularly relevant in very young children, as young as two years old (41), females (25) and minority groups (22, 41). For example, African-American female adolescents are at greater risk for becoming overweight and obese compared to African-American male adolescents (42), and other minority groups (19, 22, 41). This female/male disparity in the prevalence of obesity in young African-Americans has been found primarily in families with low levels of parental education (42). Moreover, childhood psychosocial factors are better predictors of overweight/obesity in later life among females than males (43).

### Adverse life events

Other psychosocial factors may also be relevant to the increase in childhood overweight/obesity. These include lack of social networks, social isolation and marginalization (9, 36), single parent families, parental unemployment (42), child neglect (44), domestic violence (45), early sexual harassment and abuse (46–50), and parental psychopathology (15, 21, 43, 51–53).

Several processes may mediate the association between early-life trauma and overweight. These include greater rates of skipping meals to lose weight, problematic eating-related preoccupations and behaviors, greater use of food in response to stress, and reduced physical activity (15, 45, 46). Furthermore, greater levels of depression (49, 54) and hypothalamic-pituitary-adrenal axis dysregulation (49), both considered to increase the risk for overweight/obesity, are found in girls with a history of childhood abuse in comparison to controls. Interestingly, severity of early childhood trauma remains a significant predictor of later overweight/obesity even when controlling for the influence of minority status (49) and of trauma-related psychiatric comorbidity (50).

### Media exposure and food advertising

Children in the US and other industrialized countries are exposed to the media for an average of 3–5 h per day, which is greater than any other daily activity, except for sleeping (55–57), far exceeding the television-viewing recommendations of less than 2 h per day (25, 58). Additionally, the prevalence (56, 57) and severity of overweight (55) has been directly related to the amount of time spent watching television (25). Children’s television-viewing patterns are strongly associated with those of their parents (59). Several mechanisms may be involved in the association of media exposure with overweight/obesity (55, 56, 60), this association being of greater relevance in girls. These mechanisms include media viewing replacing time spent in physical activity (15); exposure to advertisements encouraging unhealthy food choices; use of popular characters to promote high-calorie foods; excessive eating while viewing television (primarily of energy-dense snacks that may promote weight gain); and exposure to distorted messages regarding the way to achieve an ideal weight (61, 62). Interestingly, some studies have shown that the effect of television-viewing time on childhood overweight/obesity may be independent of the associated decrease in physical activity, being attributed solely to the increased total energy intake during television watching (56).

Another significant contributor to the association between media exposure and childhood overweight/obesity relates to the excessive exposure to marketing and advertisements of unhealthy foods targeted toward children (63). Food marketing to children is a massive global industry, transmitted via a variety of media outlets including television, placements in toys, games, songs, movies, celebrity endorsements, cellular-telephone text and messages, and the Internet (40, 64). The conclusions of a large-scale review (40), analyzing the results of 123 studies addressing the issue of food marking and childhood overweight/obesity in USA, support the likelihood of a link among food marketing, children’s food preferences and food consumption, and weight. Since 1994, U.S. companies have introduced over 600 new food products for children, half of them being candy or gum like items, and another 25% other types of sugary or salty snacks. Only a quarter are healthier items, such as baby foods, bread products, and bottled waters (40). Fast-food restaurants, candy, cereal, snacks, and soft drinks are the food products most commonly advertised on popular children’s Web sites and in television (64–66). These products are also abundantly available around the vicinity of primary schools (63). Many food-related advertisements have been found to use the term “super-charged” or a similar adjective to describe the powerful taste or other physical properties of unhealthy and usually high calorie foods. By contrast, statements or depictions that a food product is healthy or nutritious are infrequent in commercials (67).

Media exposure and food-related marketing have consistently been shown to have negative effects for children in general (65, 68, 69), but are particularly deleterious in children who are already overweight or obese (69, 70), as well as younger children (71). Specifically, overweight children may show greater responsiveness to food branding, likely being at greater risk when exposed to messages that are highly inundated with food-related contents (35). Moreover, exposure to food advertisements may produce a substantial increase in energy intake among children, this is evident in the increase in the amount of food intake (in particular intake of high-fat snacks) following exposure to food-related commercials (69). Still, the greatest increase in consumption of calorie-dense snacks in these conditions is found in obese children. In addition, overweight and obese children show greater preference for energy-dense foods in comparison to normal weight children (60).

It is noteworthy that whereas many studies highlight the detrimental influence of advertisements on food-related habits of children, Kopelman et al. (72) have shown that although 9–11-year-old children do demonstrate high brand logo recognition abilities, these are not associated with unfavorable eating behaviors, food knowledge, or food preferences. Still, this study has not controlled for the participants’ BMI, shown elsewhere to be associated with sensitivity to food advertising. Moreover, low SES has been found in this study to represent the most influential contributor to poor food choices among children in this study. Accordingly, further research is required to further elucidate the influence of exposure to media in general and to food advertising in particular on children’s eating behaviors and weight.

### Parental attitudes and behaviors

One of the major psychosocial contributors to childhood overweight/obesity is the food-related family environment. In particular, parental attitudes and behaviors play a critical role in the development, maintenance, and prevention of childhood overweight/obesity. Moreover, one of the most important predictive factors of childhood overweight/obesity is parental (primarily maternal) overweight. This has been found in both industrialized (21, 34) and non-industrialized (28, 29, 73) countries.

#### Parental feeding strategies

Another major factor associated with childhood overweight/obesity relates to parental feeding strategies, first and foremost the inclination of parents to control and restrict food from their overweight children. While this practice is highly common, it is particularly harmful in increasing the risk of later overweight/obesity (21, 74–76). This is associated with the likelihood of restrictive approaches to feeding to interfere with satiety cues and with the natural ability of children to control and regulate their food intake. Restriction also may increase the risk for opportunistic snacking, preference of unhealthy food, and dysregulated eating in the absence of hunger. This, in turn, may unfavorably affect the capacity of children to consume moderate amounts of food once restriction is no longer present (74, 77, 78), thus increasing the risk for later overweight/obesity (79, 80). Indeed, highly directive restrictive strategies have been consistently associated with higher weight status (81, 82). The occurrence of restrictive feeding patterns is likely the result of a bidirectional relationship between children’s weight and parental response. Accordingly, parents are more likely to restrict food from overweight/obese children, which in turn increases their risk of weight gain, further exacerbating this vicious cycle (83).

An alternative strategy associated with increased risk of overweight/obesity relates to parental pressure to eat (33, 84, 85). Up to 30% parents may mistakenly assume that their normal weight children are eating too little despite appropriate food consumption (86). Similarly, mothers may have a particularly difficult time withholding food when a child claims to be hungry, despite having eaten. Providing ample nourishment is often conceived as an important and emotionally rewarding aspect of parenting, likely being difficult to relinquish (86).

From a different perspective, several prospective longitudinal studies have assessed the influence of different parenting styles, authoritarian (high demandingness/low responsiveness), authoritative (high demandingness/high responsiveness), indulgent (low demandingness/high responsiveness), and uninvolved (low demandingness/low responsiveness) on eating behaviors and weight. These studies have consistently shown that the authoritative parenting style has the most beneficial effect on children’s eating behaviors and weight (78, 87–89). In the feeding domain, authoritative parents encourage eating by using predominantly non-directive and supportive behaviors, including reasoning and complimenting. Most importantly, authoritative parenting style allows children to have control over their food choice. It nevertheless implies that although parents tend not to restrict available food, they do control the availability of food at home. This strategy has been found to maximize the consumption of healthy food and to minimize the consumption of unhealthy food (76, 84). Parental feeding styles have been associated with their educational level. For example, mothers with higher education may exert significantly greater control over their child’s feeding, and tend to be significantly less emotionally reactive while feeding their children than mothers with lower education levels (90, 91).

### Sleep deprivation

Recent widespread changes in the sleep pattern of children and adolescent may also play a significant role in the recent increase of pediatric obesity (92–94). Curtailment of sleep duration has become a widespread habit and a hallmark of modern society. According to the annual surveys conducted by the American Sleep Foundation, only 20% of adolescents get the optimal amount of sleep they need (94). Recent physiological evidence has shown a link between lack of sleep and the development of obesity (95). In general, decreased sleep leads to fatigue, negative mood, and overall psychological distress, potentially altering or dysregulating eating patterns.

Several mechanisms may play a role in the recent decrease in sleep quality and its impact on the eating patterns of children and adolescents. First, greater access to media and video games interfering with sleep time may lead to increased food consumption in an effort to increase alertness. Second, there is a tendency to snack simultaneously while engaging in media exposure activities (96). Third, the difficulties of parents in setting limits and good sleep habits for their children can also have an impact on the quality and amount of sleep. One of the most significant factors relating to sleep patterns in adolescence is the extent to which parents supervise bedtime schedules. Thus, as adolescents mature, their parents become less involved in bedtime decisions (97, 98). Adolescents tend to sleep more at night over the weekends, based on the premise that they can make up for lost sleep time during the week. However, this premise is considered only partially correct, and research has shown that there is no satisfactory compensation for the sleep deprivation experienced during the week.

Increased environmental expectations and pressures stemming from academic demands, social and sport activities, part-time employment, and preoccupation with the internet, television, and cell phone may also lead to reduced sleep and the resulting changes in eating patterns. Still, some after-school activities may influence sleep patterns more than others. For example, whereas homework only partially involves later bedtime, extracurricular activities, especially sports, have a more detrimental impact. In addition, part-time employment after school likewise increases sleep deficiency among those who work more than 19 h a week (97, 99).

Another factor affecting sleep deficiency during the weekdays is the early start time of school. A recent study conducted in Israel (99, 100) has shown that delaying the opening of school by 1 h (from 7:30 to 8:30) results in 51 more minutes of sleep time without influencing sleep onset time. Similar findings have been found in studies conducted in the United States (101).

To summarize, many children and adolescents do not achieve sleep time that is sufficient according to accepted guidelines. As a result of insufficient sleep, children and adolescents may suffer from mood, attention, academic, and memory deficits as well as from alterations in eating patterns, including greater risk of overweight/obesity. Because of the health implications associated with insufficient sleep in children and adolescents, improving sleep quality is the responsibility of parents, educators, sports coachers, older children, and adolescents themselves. Parents must exert some level of control over their adolescents’ daily and evening schedules, even at the cost of interfering with age-appropriate independence, to ensure that they have sufficient opportunity for adequate sleep. Educators must strive toward appropriate school starting time and moderate homework loads. In addition, the scheduling of extracurricular activities, particularly sports, should promote, rather than detract from adequate sleep (102).

## Intervention Programs

The recent increase in childhood overweight/obesity places a significant burden on physical, psychological, and social health. It is therefore essential to alter those environmental and behavioral factors that may potentially increase the risk of childhood overweight/obesity, while simultaneously increasing those factors potentially protecting against it (103). Interventions for childhood overweight/obesity generally aim to modify lifestyle behaviors such as food intake and physical activity. Despite great efforts to alter these behavioral factors, most interventions have been found ineffective (104, 105).

A variety of factors may account for these negative results. First, interventions that target only children and do not involve parents or the larger family structure are not likely to be successful (34, 106). Involving parents in the attempt to prevent or reduce childhood overweight is of vital importance (107), as they are the primary figures in adjusting their children’s food and activity environments (34). Accordingly, the manner in which parents feel and act with respect to eating, food consumption, and physical activity may have an important role in shaping similar perceptions and behaviors in their children (84, 108, 109). In addition, overweight/obese children often feel that initiation of a behavioral change requires the active intervention of a role model, primarily a parent, whereas delayed parental recognition with respect to the necessity of behavioral change may interfere with action taking (110). Indeed, between 30 and 50% of parents of overweight and obese children do not attempt to change their child’s eating habits and weight (111–113). Furthermore, parents of 4–11-year-old obese children have been found to exhibit lower levels of parenting self-efficacy in comparison to parents of normal weight children (114), highlighting the even greater necessity of involving them in the interventions. This may also be the case in older overweight/obese children, as their parents may feel incompetent in their ability to change the eating-related behaviors of their children and to serve as role models (115).

Second, programs targeted at overweight/obese children often focus solely on weight reduction with little emphasis on psychosocial influences such as media or peer pressure (116). Third, programs often fail to address the manner in which overweight children perceive their own weight. Although accurate awareness of one’s overweight may decrease self-esteem, accurately perceiving oneself as overweight/obese may increase the motivation for changing maladaptive lifestyle behaviors (105). This is of particular relevance as overweight and obese youth are more likely to misperceive their weight compared with their normal weight peers (105).

### Familial involvement

#### Primary prevention

The entire family should be involved in both primary prevention programs as well as in interventions targeted at children who are already overweight or obese. Primary prevention programs attempt to promote healthy eating and weight-related behaviors and to increase physical activity in younger individuals at large. This can be done in focus groups that include only parents, or both parents and children (107). Drawing definite conclusions about the efficacy of such primary prevention programs or the factors affecting it is currently still limited; this because they have usually involved only a relatively small number of families, and had no long-term follow-up assessments (107). Moreover, McGarvey et al. (117) suggest that whereas the combination of psychoeducation with behaviorally targeted changes may affect the attitudes and behaviors of parents, the effect of these parental changes on the behavior of the overweight child is limited. By contrast, others (118) conclude that despite the great variability in the design, duration, and outcome assessments of primary prevention studies carried out in the 1990s, these programs may have the potential to reduce the rate of overweight in some populations.

In a more recent randomized controlled trial (RCT) involving the entire family, Fulkerson et al. (119) examined the feasibility of a pilot parent/child overweight/obesity prevention program. The program included five sessions consisting of interactive nutrition education, taste testing, cooking skill building, parent discussion groups, and hands-on meal preparation. Although significant favorable differences have been found in comparison to control families at post-intervention with respect to healthy food choice, many of these changes have not been maintained at 6 months follow-up, and no differences in weight reduction have been found in research vs. control children.

Most researchers agree that creating a more positive environment surrounding food has to involve the entire family (33). Neumark-Sztainer (109) emphasizes that parents are often confused with respect to the types of messages they should send, or not send, to their children regarding eating and weight. She outlines several strategies for parents to deal with potentially harmful eating-related societal pressures and to decrease their child’s risk for becoming overweight. The first strategy involves modeling health behaviors, emphasizing that parents should model behaviors they wish their children to emulate, particularly engaging in healthy eating of a variety of foods and exercising at an appropriate level. Additionally, parents should refrain from behaviors and comments encouraging unhealthy eating- or weight-related behaviors such as dieting or excessive exercising. The second strategy suggests that parents should provide an environment that makes it easy for their children to make healthy choices. Thus, instead of trying to restrict and control their child’s dietary intake, parents should provide a home environment that includes an abundance of fruits and vegetables, encourages family meals, and has a minimal number of televisions (109). Others (120) have suggested increasing children’s water consumption as a part of a healthy eating environment. The third strategy involves the promotion of adaptive attitudes and behaviors rather than focusing on weight. It encourages parents to assist their children in developing an identity that goes beyond physical appearance to non-appearance-related traits and accomplishments, and to moderate their interest and preoccupation with weight and dieting. The fourth strategy aims to provide a safe base in a weight-obsessed environment, teaching parents how to support their children when being teased outside home about their weight.

#### Secondary interventions

Interventions designed to target children who are already overweight/obese are also found to be more effective when involving parents. RCTs performed by Epstein et al. (121) provide evidence that behaviorally oriented treatment programs targeted to reinforce a change in eating behaviors and weight loss in both the parents and their overweight child show better results after a follow-up period of 10 years when compared to treatments focusing solely on changing the child’s eating behaviors and weight. Similarly, Golan et al., conducting several RCTs involving both parents and children (122–124), or parents only (124, 125), have concluded that both programs are more effective than targeting children. Still, involving only the parents with a family health-centered approach has been associated with greater weight loss and higher consumption of healthy foods in overweight children compared to treatment strategies involving both parents and children. Thus, involving the parents, either exclusively or with their children, has shown overall beneficial long-term results not only for the overweight child but also for the entire family, both with respect to their eating- and weight-related behaviors and their overall functioning (2).

In addition, parents may play a critical role in the modification of behaviors surrounding media consumption. It has been noted that greater television and other media exposure in children is directly related to overweight/obesity. Therefore, parents have a role in altering their children’s media viewing habits and, in turn, their sedentary-related behaviors. Some cross-sectional (57) and prospective longitudinal studies (126) have shown that parents of young children have the ability to reduce their snacking while watching television, to close the television when no one is watching, and to take the television out of the child’s room, although actual television consumption time may not be significantly reduced. While these findings may seem promising, most studies have not included long-term follow-up, and their effect on eating-related behavioral change and weight reduction in overweight children is limited at the present stage.

### Physical activity interventions

Promotion of healthy physical activity is an integral part of any program aiming toward the reduction of childhood overweight/obesity (127). Research has repeatedly shown that being inactive or minimally active during childhood and adolescence, as opposed to performing adequate activity during these critical developmental periods, may considerably increase the risk of developing overweight/obesity not only in childhood but also in later life. In addition, greater frequency of physical activity also appears to be related to improved mental health status in overweight/obese children (127).

The results of studies assessing the efficacy of interventions aimed at increasing physical activity are not consistent. Some studies have shown that increasing children’s activity does reduce weight gain (128). An RCT involving parents and children in a weekly 2-h fun physical activity over a 2-month period, has found significant improvement in children’s waist circumference, BMI, body composition, physical activity level, sedentary activities, cardiovascular fitness, and self-esteem in comparison to a control waiting-list group both at post-intervention and at a 12-month follow-up (129). Similarly, engaging siblings (130), peers (131), and the entire family (132) in physical activity, combined with parental support and modeling, may have a significant beneficial effect on the physical activity of overweight children, and on their BMI.

By contrast, an RCT showed no change in BMI following a physical activity intervention in Scottish pre-school children at 6- and 12-month follow-up despite increasing the children’s physical activity levels (133). Methodological considerations, including the amount and quality of the physical activity introduced and/or the training methods and outcome measures used, may account for these inconsistencies (134). Specifically, not only weight loss but also maintenance of weight should be considered a positive outcome.

### School-based prevention programs

Peer influence and the entire school environment are major contributing factors to the development of food, physical activity, and health-related preoccupations and behaviors among the students. Indeed, numerous school-based interventions have been planned to intervene in the influence of these factors on childhood overweight/obesity. Still, the efficacy of these programs remains inconsistent.

Several interventions, mostly (135–138) but not all (139, 140), assessed by controlled designs have aimed to improve the food environment at school. These include nutrition and education (137), the delivery of frequent and systemized positive messages about eating healthful food and modeling fruit and vegetable consumption (135, 138), changing the types of foods sold and served at school according to accepted guidelines (136–141), reducing the amount of consumption of carbonated drinks (139), promoting healthy physical activity at school (127, 136, 138, 141) and healthy social marketing (137), and parent outreach (137, 138). These changes have yielded a positive impact not only on children’s knowledge and attitudes toward exercise and healthy eating but also on their eating behaviors, weight status, overall lifestyle, and mental health. An additional approach, “edutainment” – combining the active participation of youngsters and popular celebrities in a musical (active entertainment) with a lifestyle change workshop (education) initiated in Israel, has shown an improvement in the participants’ physical activity, eating patterns, and attitudes toward dieting (142).

Conversely, other studies have shown that school-based overweight/obesity prevention programs have not altered school nutrition and physical activity (25, 134, 143–145), specifically if applied in rural areas with lower SES (145). A comprehensive review article associates these negative findings, at least in part, with the lack of assessment of eating disorders and other psychiatric disorders in most of these studies, and with inadequacy of the measurements used to assess change in overall well-being, functioning, and other relevant psychological variables (146).

Cole et al. (104) reviewed school-based interventions found to be effective in reducing BMI in overweight children between the ages of 4 and 14 to better understand the factors associated with these positive findings. They concluded that effective school-based interventions should use a combination of multiple treatment modalities incorporating elements based on social cognitive and motivational factors. These include focusing on readiness for change, self-evaluation and self-monitoring, peer support, observational learning, appropriate social setting (the class), and accepted modeling figures (teachers, school nurses, and other familiar facilitators).

Many states in the US have implemented regular screening of overweight/obesity, by measuring weight and height in schools. The rationale underlying this strategy is that sharing weight status information could increase parental awareness of the child’s weight, and motivate them to take appropriate actions to address potential weight problem (147). The efficacy of such programs is still a matter of debate. Moreover, the implementation of such programs raises serious concern over their potential hazards. Eating disorders experts are particularly concerned about the potential of such screenings to induce disordered eating- and weight-related preoccupations and behaviors in vulnerable individuals. Another focus of concern relates to their possible unfavorable influence on the well-being of the overweight/obese child at school. Thus, whereas the majority of middle school students do not report discomfort with school-based weight screening, more than a third of overweight students are uncomfortable with it (147). Similarly, parents of overweight/obese children may be less likely to be supportive (148) of school-based screenings compared to parents of children of a healthy weight (149), who are also not always in favor of them (150).

While most school-based interventions target food and physical activity environment, they often fail to address weight-related teasing and stigma, which has shown to be associated with increased overweight and overall psychological distress (9, 151, 152). This is even of more concern because although providing external explanation for overweight/obesity at school can change children’s cognitions about obesity, it is less likely to improve their overall negative attitudes (9).

### Other interventions

A recent RCT (153) assessed the efficacy of adding a newly developed computerized device, the Mandometer, to a standardized life style modification program vs. the use of this program alone in 106 newly referred young obese people aged 9–17. The aim of this device is to provide real-time feedback during meal time to slow down the speed of eating, hence inducing greater satiety responsiveness, and to reduce total food intake. Participants using this device have shown significantly lower meal size at the end of treatment, leading in turn to significantly lower BMI standard deviation scores (SDS) and lower mean body fat in comparison to the control condition. The post-treatment reduction in portion size has not been associated with a significant change in perceived satiety at the end of meal compared with the satiety levels at study entry, indicating that the participants have felt as full while consuming less food. The reduction in BMI SDS has been maintained 6 months after the end of treatment. The authors further suggest that although it is unlikely that a specific eating pattern is typical for all obese individuals, this method may be specifically tailored for young people eating large portions very fast. The weakening of the effect of reduced portion size during treatment suggests that intermittent short periods of retraining may be necessary to maintain maximum benefit.

### Intervention at the public policy level

The interventions reviewed above suggest that programs aiming to prevent, or at least reduce, the ongoing rise in childhood overweight/obesity should be multi-faceted and focus on both the family and the social environment of the overweight/obese child (34). Still, interventions at the local level are likely not sufficient, calling for the implementation of large-scale interventions on public policy level (64, 71, 154). Such interventions should be multimodal, focusing on several factors: the influence of the media on the child and the family, the overall reduction in physical activity and increase in sedentary activities in current Western societies, the availability of unhealthy snacks and soft drinks in school and at home, the change in portion sizes in restaurants, and the increase in marketing of food products with a high-fat content.

Along these lines, Schwartz and Brownell (155) suggest that public policy prevention programs should relate to pediatric overweight/obesity as a consequence of a “toxic environment” rather than as the result of the population failing to take enough “personal responsibility.” Thus, in order to make progress in reducing the incidence of childhood overweight/obesity, policy makers should change the view of overweight/obesity from the medical model (which focuses on the individual) to a public health model (which focuses on the population at large). Furthermore, these programs should be aimed for young children, because in this age interventions are most likely to successfully modify risk factors for later overweight/obesity, including the food environment at home and at school, decreased physical activity and greater sedentary activity, peer teasing, and the influence of food advertisements and marketing (156, 157).

Based on prospective longitudinal exploratory research, the American Academy of Pediatrics suggests the following recommendations for action on a policy level: regulation of food advertisements targeted at children; expanding public education to promote healthy eating and exercise; introducing healthy food at school meals; incorporating messages about healthy lifestyles into television programing scripts; reducing exposure of children to the media, while at the same time promoting media literacy; implementing multiple treatment modalities in school-based interventions addressing childhood overweight; and encouraging pediatricians and other health professionals to assess media exposure when treating children and families (58). It is further recommended that children should participate in a minimum of 30–90 (mean 60) min of physical activity daily and spend no more than 2 h a day using electronic media for entertainment (25, 59, 158).

Another area requiring regulation at the public policy level is related to food marketing aimed at children, as it has been shown to impact unhealthy food choices (71, 159). Specifically, research suggests that restrictions at the public policy level may be particularly effective for this purpose (160). Currently, more than 50 countries around the globe have implemented laws that regulate television food advertising aimed at children (159). In addition, marketing-related regulatory codes have been advanced in recent years by the IOTF (10) and the United Nations System Standing Committee on Nutrition (161). It is recommended that actions to reduce food marketing to children be mandatory, guarantee commercial-free childhood settings, and include all forms of media (71, 159). These actions should further be evaluated, monitored, and enforced on a routine basis (71). Future research should focus on the impact of these regulations on food and activity choices among children and families.

An additional area requiring interventions on a public policy level is the providing of resources, guidelines, and psychoeducational training about childhood overweight/obesity to health care providers. This is because primary healthcare providers may often lack adequate knowledge, and/or harbor prejudicial attitudes toward pediatric overweight/obesity (162–166). For example, general practitioners and practice nurses may feel that overweight/obesity in children is a familial or social but not a medical problem (167). Additionally, trained personnel often feel lack of comfort, practice, and confidence when faced with issues related to childhood overweight/obesity (163). As a result, primary health care providers may refrain from addressing vital obesity-related issues, particularly the child’s activity level and television-viewing habits (168). Further, in some cases, even the child’s BMI is not routinely measured (162, 169). In other studies, over 35% of primary care providers have never discussed fast food, television, or candy use with parents of overweight/obese children, and 55% have never discussed exercise (164). Finally, health care professionals are less likely to accurately assess history and risk factors related to overweight/obesity in pre-school age children in comparison to adolescents (168).

Still, in other studies, primary care providers have been found to have adequate knowledge about factors influencing the risk of overweight and obesity in children, but have nevertheless often felt that many barriers may preclude adequate interventions (170). Thus, in the opinion of many health care providers, providing diet and exercise advice may not have adequate impact on a child’s weight (167) because of the low compliance of parents and children (163, 170). Still, the low rates of success in the attempts of health care providers to intervene with overweight/obesity, may actually be related to the limited training and knowledge of professionals regarding the way in which to communicate with parents and children about these issues (167, 171). Other likely barriers for adequate interventions include culturally related issues and time constraints (167).

It is thus of immediate relevance to initiate programs designed to improve the knowledge, attitudes, sense of self-efficacy, and commitment of primary caretakers treating overweight/obese children (162–164, 172). Research indicates that providing adequate training to health care practitioners does enhance their knowledge and communication skills (173) and increases their sense of self-efficacy (166). Supporting these findings, it has been shown that overweight/obese children whose parents and primary caretakers received training in motivational interviewing techniques had a greater reduction in their BMI in comparison to control participants (155).

Interestingly, school and kindergarten staff may possess more positive attitudes toward childhood obesity than primary health care providers. For example, in one study, kindergarten teachers were both aware of and concerned about overweight/obesity among children, felt that encouraging healthy eating and active play were part of their core responsibilities, and worked closely with parents. They further identified a range of helpful educational resources and professional development opportunities to deter parents from sending unhealthy foods to school with their child, and to assist them in deciding whether (and how) to discuss their concerns about a child’s weight with the parents (174).

## Conclusions, Limitations, Recommendations, and Future Directions

A dramatic rise in childhood overweight/obesity has been shown in recent decades to occur in both industrialized and non-industrialized countries. This increase has been most pronounced in nations that are in transition from more traditional to more industrialized lifestyles (175).

Genetic as well as psychological, environmental, and socio-cultural factors are thought to account for this rise. This paper has set its aim to review the role that gender, SES, cultural and familial background, social isolation, media exposure, changes in food preferences, and reduction in physical activity may play in the recent increase in the rates of childhood overweight/obesity. For this aim, we conducted an extensive comprehensive literature search about the psychosocial factors likely influencing the development and maintenance of overweight and obesity in children and adolescents and about potential primary prevention programs and secondary interventions.

Several limitations of this review should be addressed. First, we included in our review not only findings of RCTs or controlled designs but also of open studies. This was done in an attempt to provide a more clinically oriented review to broaden the knowledge and understanding of professionals in an area that may suffer from lack of adequate knowledge and prejudicial attitudes, and to create a place for discussion, rather than providing only evidence-based recommendations and tools. Second, similar to many other studies in this field, we were not always able to distinguish between findings and recommendations related to overweight vs. obesity. The specific time frame of this review, from 1991 onward, is in keeping with our previous review on psychological perspectives of childhood overweight and obesity (176).

To conclude, it is critically important to consider psychosocial factors in the prevention and/or reduction of childhood overweight/obesity. Findings from prevention studies emphasize two important points. First, prevention programs should be multidisciplinary; combining the knowledge of experts from different professions, taking into consideration the important role of the family environment (122) and relevant influential social organizations, with a particular emphasis on the child’s school environment (103). Second, effective change is unlikely without large-scale programs carried out on a public policy level (154).

Furthermore, in line with the findings emphasized in this review, prevention programs should take into consideration the developmental stage of the child and his/her capacity for change, his/her personality structure and psychopathology, the SES, and the functioning of the family system, as well as issues related to ethnicity and cultural background to potentially increase their efficacy. Interventions should be developed and piloted in consultation with the target community, in particular in the case of ethnic minorities, to improve acceptability and compliance (43, 157, 177). It has increasingly been acknowledged that individual behaviors and lifestyles factors, such as food choices or child-feeding practices, are responsive to the socio-cultural and ecological contexts in which they are practiced (37). For that purpose, programs should utilize appropriate settings such as the child’s home, post-natal clinics, day care centers, schools, community centers, and places of worship, to provide an opportunity for the involvement of family members and other significant figures (177). They should promote and implement changes in lifestyle, eating behaviors, physical activity, and media literacy. Specifically, multidisciplinary programs combining experts of different professions, advocating health promotion approaches, and communicating research findings to policymakers may be a promising population-based initiative to prevent, or at least reduce, the risk of childhood overweight/obesity (154, 157, 177, 178)

Finally, further recommendations for the prevention and/or reduction of childhood overweight/obesity at the public policy level rand for future research relate to: 1. Provision of education to primary health care professionals about childhood and adolescent overweight/obesity to increase uniformity of assessment and to improve caretakers’ knowledge, awareness, overall attitudes, and sense of self-efficacy in managing these problems. 2. Inclusion of health promotion and media literacy programs in schools’ curricula. 3. Regulation of food/physical activity advertisements targeted at children. 4. Expanding public education and environmental changes to promote healthy eating and increased physical activity, and to reduce sedentary behaviors. Emphasis should be placed on physical activity also outside of school (179), and on provision of resources focused on increasing the availability of neighborhood outdoor space (127, 180). 5. Promotion at the public policy level of legal, legislative, and industry-based activities to better regulate the food provided to young people at school and other institutions. 6. Preventive interventions at the public policy level should coordinate all parties involved, including the children, their families, schools, primary care professionals, government agencies, industry, and media. 7. Emphasize the role of parents on all levels of designing prevention programs.

## Conflict of Interest Statement

The authors hereby confirm that this paper was not supported by any external funding from public or private agencies. There are no known conflicts of interest.
